# Effect of Nano Ni Particles on the Microstructure and Thermophysical Properties of Sn–Bi–Zn Heat Transfer and Heat Storage Alloys

**DOI:** 10.3390/ma16155325

**Published:** 2023-07-28

**Authors:** Qingmeng Wang, Xiaomin Cheng, Xiuli Wang, Tao Yang, Qianju Cheng, Zhi Liu, Zean Lv

**Affiliations:** 1 School of Mechatronics and Intelligent Manufacturing, Huanggang Normal University, Huanggang 438000, China; wangqingmeng@whut.edu.cn (Q.W.); wangxl2022@hgnu.edu.cn (X.W.); yangtao@hgnu.edu.cn (T.Y.); qianju1990@163.com (Q.C.); lz3839@126.com (Z.L.); lvzean@163.com (Z.L.); 2 School of Materials Science and Engineering, Wuhan University of Technology, Wuhan 430070, China

**Keywords:** nanoparticles, microstructure, thermophysical, Sn–Bi–Zn

## Abstract

The specific heat capacity plays a crucial role in influencing the heat transfer efficiency of materials. Considering the relatively low specific heat capacity of metals, this study focuses on investigating the impact of second-phase nano Ni particles on the microstructure and thermophysical properties of the alloy matrix. The alloys’ phase compositions and microstructures were examined using X-ray diffraction phase analysis (XRD), electron probe micromorphology analysis (EPMA), and X-ray fluorescence spectroscopy (XRF). Furthermore, the thermophysical properties of the alloys were comprehensively analyzed through the employment of a differential scanning calorimeter (DSC) and the laser flash method (LFA). The addition of second-phase nanoparticles significantly increased the specific heat capacity of the alloy in the liquid state; however, the phenomenon of nanoparticle agglomeration diminishes this improvement. The analysis of the specific heat enhancement mechanism indicates that ordered states are formed between the second-phase solid nanoparticles and the melted metal in the liquid state. With the increase in temperature, the destruction of these ordered states requires additional heat, resulting in the increase of specific heat capacity.

## 1. Introduction

Currently, countries worldwide have implemented various policies to promote the extensive development of new energy sources and reduce reliance on fossil fuels. These policies aim to drive technological innovations in areas such as solar thermal utilization and enhance the overall quality of energy production [1]. In the realm of solar thermal power generation technology, industrial applications and waste heat recovery and utilization, as well as building heating and hot water systems, heat transfer materials, and heat storage materials, play a crucial role. Research on heat storage and transfer mechanisms serves as the foundation for achieving efficient energy utilization [2,3,4]. Conventional heat transfer and storage materials, including molten salt and heat transfer oil, possess several drawbacks, such as high-pressure leakage, decomposition, deterioration, and strong corrosiveness [5,6,7,8,9]. Conversely, multi-component alloys have emerged as promising alternatives for high-temperature heat transfer and storage fluids in recent years. These alloys exhibit desirable properties, including high heat storage density, a wide operating temperature range, good fluidity, and high thermal conductivity. Consequently, they have garnered significant attention within the fields of solar thermal power generation and heat transfer and storage material research [3,10,11,12].

On the other hand, metals have a relatively low specific heat capacity, which significantly restricts the heat transfer and storage capabilities of liquid alloys [13,14,15]. Enhancing the specific heat capacity in a targeted manner would greatly improve power generation efficiency. Current research findings indicate that the addition of nanoparticles can enhance the specific heat capacity of heat transfer fluids, with a focus on molten salts and heat transfer oils [16,17,18]. For instance, Ahmed et al. [19] doped graphene with a mass fraction of 0.1% into a multi-component nitrate, resulting in an increase in specific heat capacity from 1.26 J·g^−1^·K^−1^ to 1.46 J·g^−1^·K^−1^. Huang et al. [20] investigated the impact of different mass fractions (0–15%) of MgO nanoparticles on the specific heat capacity of nitrate. The results revealed that the specific heat capacity of Solar salt initially increased and then decreased with the increasing mass fractions of MgO nanoparticles. At a doping mass fraction of 10%, the specific heat capacity of MgO/Solar salt reached 3.62 J·g^−1^·K^−1^, demonstrating the highest enhancement effect. Furthermore, Han et al. [21] doped binary Solar salt with Al_2_O_3_ nanoparticles ranging in size from 80 to 1000 nm, with a mass fraction of 1%. The study examined the effect of Al_2_O_3_ nanoparticle size on the specific heat capacity of nitrate, and the results indicated that the most significant enhancement effect was observed with Al_2_O_3_ nanoparticles of 135 nm in diameter, increasing the specific heat capacity from 1.27 J·g^−1^·K^−1^ to 1.52 J·g^−1^·K^−1^. These research findings validate the efficacy of nanoparticles in enhancing the specific heat capacity of heat transfer and storage materials. However, considering the differences in the crystal structure, microscopic morphology, and bonding methods between metals and their alloys with molten salt or heat transfer oil, further research and verification are required to investigate the experimental methods and enhancement mechanisms involved.

In this paper, the alloy developed in the previous stage is utilized as the matrix, while second-phase nano Ni particles are incorporated for modification [22]. The controllable preparation and performance regulation methods of cross-scale composite heat transfer and heat storage materials were investigated, alongside the exploration of the mechanisms by which nanoparticles influence heat storage and transfer.

## 2. Materials and Methods

According to the design composition, the corresponding grams of metal raw materials are put into the small melting furnace in turn, and the temperature is set to 350 °C. In order to prevent the oxidation of metal elements during the smelting process, an inert protective gas is added, while the mixture was continuously stirred to prepare a homogeneous Sn–Bi–Zn matrix alloy. The alloy matrix and the second-phase nano Ni particles are mixed and smelted by powder metallurgy, and the particle size is 40 nm. Compositional analysis was performed using X-ray fluorescence (XRF, Axios advanced). Table 1 shows the actual composition ratio of all alloy samples.

The phase transition temperature and specific heat capacity data of the alloy samples were obtained by the sapphire method DSC (DSC, STA449C/3/G), with a sample mass of about 20 mg, placed in an aluminum crucible. To protect the normal use of experimental instruments, a perforated lid is placed on the aluminum crucible. In the sapphire method, the heat flux is measured through an empty pot, an aluminum pot with sapphire, and an aluminum pot with sample preparation. Then, using these three results and the specific heat capacity of sapphire, the specific heat capacity of each sample was calculated. According to testing standards ASTM E1269-11 [23], the experimental heating rate was 5.0 °C·min^−1^, and the temperature range was set from room temperature to 300 °C. The accuracy of specific heat measurement should be ensured to two decimal places. The thermal expansion coefficient of the alloy samples was detected by a thermal expansion analyzer (DIL 402C, Thermo Fisher Scientific, Waltham, MA, USA). According to testing standards ASTM E831-06 [24], the heating rate was 2.0 °C·min^−1^, the temperature range was 30–80 °C, and the sample size was 5 × 5 × 20 mm.

The thermal diffusivity of the alloy samples was measured by the laser flash method (LFA 457) at 50 °C, and the sample size was set to 2.5 × 10 × 10 mm. Combined with other experimental data, the thermal conductivity of the alloy can be calculated by the following formula:

(1) $$\lambda =\alpha \cdot \rho \cdot c_{p}$$

where α is the thermal diffusivity, ρ is the density, and c_p_ is the specific heat capacity at constant pressure. The density of the alloy at high temperature was calculated from the Equation:

(2) $$\rho =\rho_{0}{\left(1+\Delta L/L_{0}\right)}^{-3}$$

where ΔL/L_0_ is the relative elongation and ρ_0_ is the density of the sample at room temperature measured using the Archimedes method.

The phase composition of the samples was tested and analyzed by an X-ray diffractometer (XRD, D8 ADVANCE, Bruker, Billerica, MA, USA) and X-ray fluorescence spectrometry (XRF, Thermo Fisher Scientific, Waltham, MA, USA). The microstructure of the alloy was observed by electron probe microanalysis (EPMA, JXA-8230, JEOL, Tokyo, Japan), and the alloy composition was analyzed by an energy-dispersive X-ray spectrometer (EDX, NCAX-ACT, Thermo Fisher Scientific, Waltham, MA, USA).

## 3. Results and Discussion

### 3.1. XRD and EPMA Analysis

**Figure 1:**
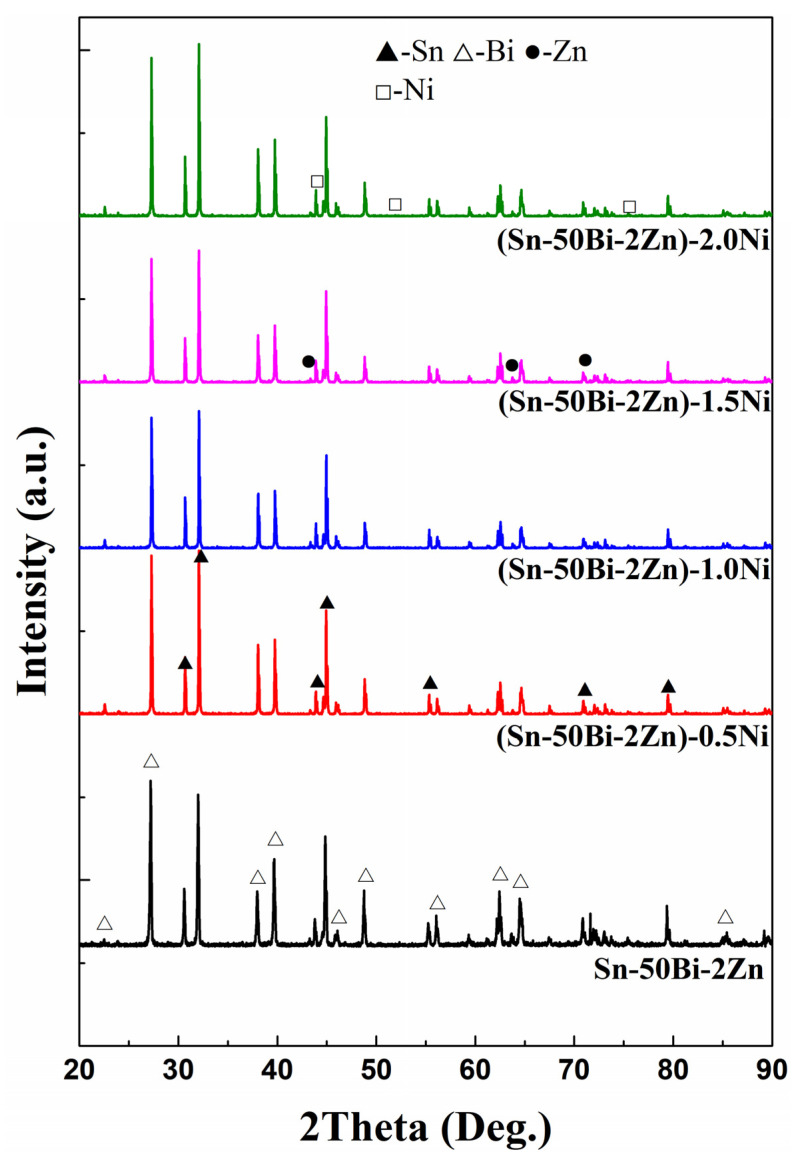
XRD patterns of A1–A5 alloys.

The X-ray diffraction (XRD) results of alloys A1–A5 are illustrated in Figure 1. Alloy A1 primarily consists of a solid solution comprising three metallic elements. However, due to the low zinc (Zn) content of only 2%, the diffraction intensity is not prominently observed. With an increase in nickel (Ni) content, the phase types and intensities of certain diffraction peaks in the A1–A5 alloys undergo changes. The alloys exhibit the presence of the tin (Sn) phase, the bismuth (Bi) phase, and Ni, while the peak corresponding to the element Zn gradually diminishes, giving way to a relatively weak Ni element peak.

**Figure 2:**
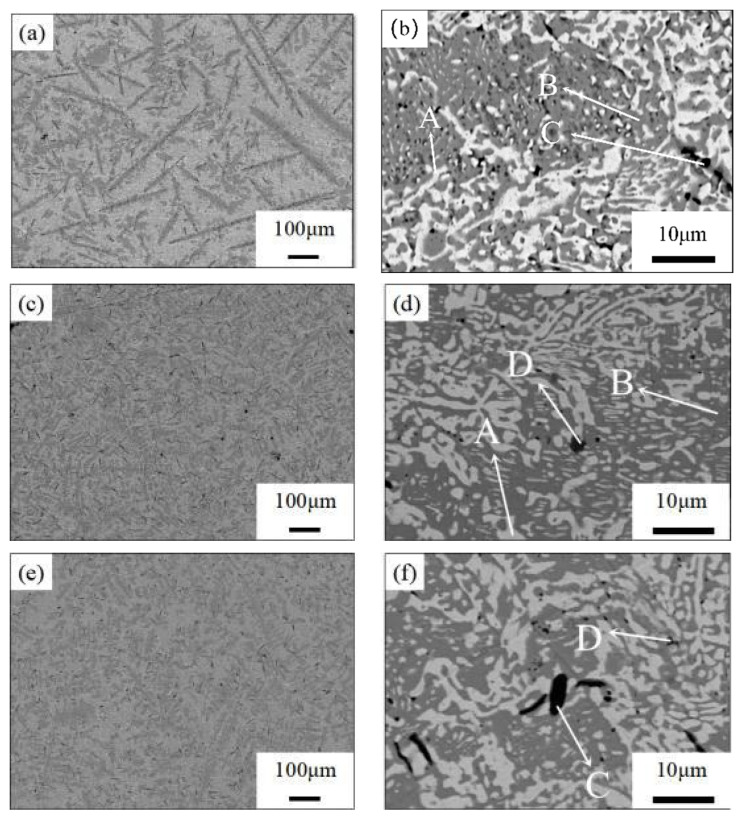

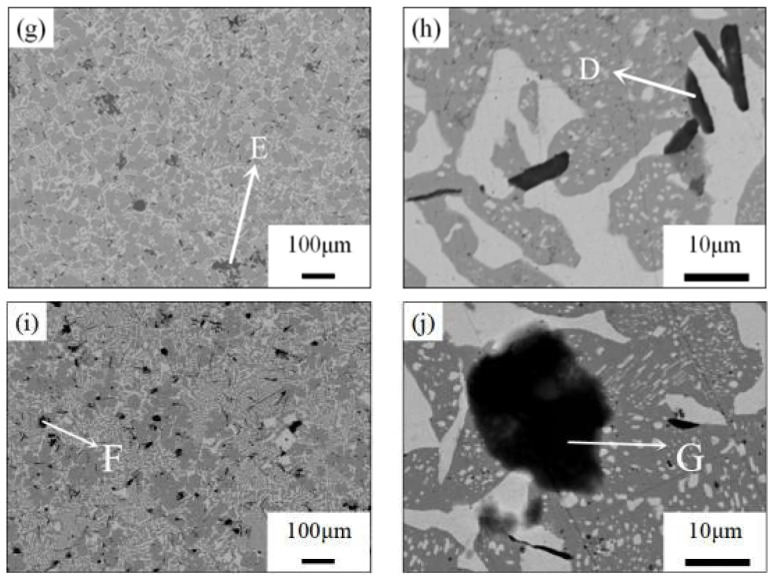
Microstructure images of the (Sn–50Bi–2Zn)–xNi alloys: (**a**,**b**) Alloy A1; (**c**,**d**) Alloy A2; (**e**,**f**) Alloy A3; (**g**,**h**) Alloy A4; and (**i**,**j**) Alloy A5.

Figure 2 presents the microstructure of the alloys, and Table 2 provides the results of the EDX analysis for the indicated regions. The microstructure images of the base alloy are displayed in Figure 2a,b, showing a eutectic structure primarily consisting of a white Bi-rich phase, gray Sn-rich phase, and dark Zn-rich phase. Figure 2c,d depicts the microstructure of (Sn–50Bi–2Zn)–0.5Ni, which is predominantly composed of a white Bi-rich phase, dark gray Sn-rich phase, and Sn + Zn phase. Point A represents a Bi-rich phase with a composition of 100 at.% Bi. Point B corresponds to an Sn-rich phase with a composition of 93.24 at.% Sn, 4.86 at.% Bi, and 1.90 at.% Zn. Point D represents the Sn + Zn phase, with an elemental composition of 59.52 at.% Sn and 40.48 at.% Zn. Figure 2e,f demonstrates that with increasing Ni content, the needle-like Zn-rich phase in the alloy decreases, resulting in a more uniform and finer microstructure compared to the matrix at low magnification. Point C represents the Zn-rich phase, comprising 20.20 at.% Sn, 16.35 at.% Bi, and 63.45 at.% Zn. The microstructure of (Sn–50Bi–2Zn)–1.5Ni is shown in Figure 2g,h, exhibiting larger and darker Bi + Ni and NiZn phases within the bulk, with the disappearance of the Zn-rich phase in the high-magnification image, and the reappearance of the bulk Bi-rich phase in the alloy structure. Point E corresponds to the Bi + Ni phase, consisting of 22.84 at.% Sn, 67.62 at.% Bi, and 9.54 at.% Ni. Point F represents the NiZn phase, comprising 16.88 at.% Sn, 12.27 at.% Bi, 50.82 at.% Zn, and 22.16 at.% Ni. The microstructure of (Sn–50Bi–2Zn)–2.0Ni is illustrated in Figure 2i,j, where the NiZn phase and Ni-rich phase are further increased, and the segregation of the Sn and Bi phases becomes evident in the microstructure. Point G denotes a Ni-rich phase with an elemental composition of 48.61 at.% Sn, 14.35 at.% Bi, 5.83 at.% Zn, and 31.21 at.% Ni. By comparing the microstructure of (Sn–50Bi–2Zn)–xNi, it can be observed that the microstructure distribution of the A3 alloy is more uniform and finer.

### 3.2. Thermophysical Properties

**Figure 3:**
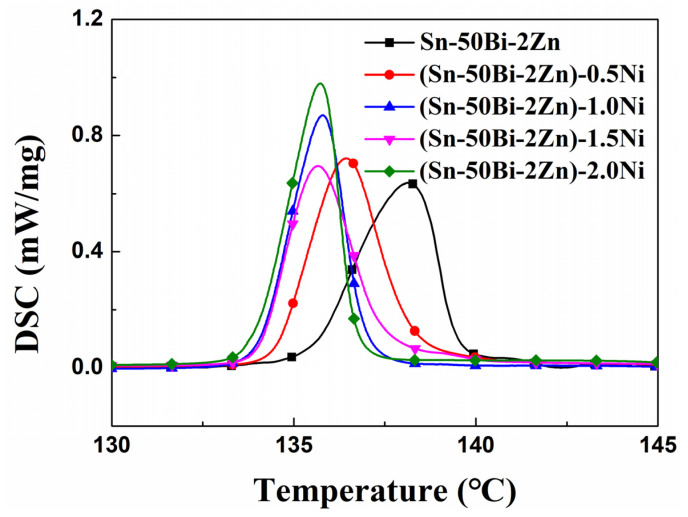
DSC curves of the (Sn–50Bi–2Zn)–xNi alloys.

The melting temperature and melting enthalpy results are presented in Figure 3, while the corresponding experimental data are listed in Table 3. The experimental results, characterized by a single endothermic peak, demonstrate the exceptional melting consistency of the alloy. The phase transition temperature of alloys A1-A5 ranges from 133.91 to 134.48 °C, with corresponding melting enthalpies of 45.24–48.14 J·g^−1^. Comparatively, the inclusion of nano-sized Ni particles has a negligible impact on the thermophysical properties of the base alloy, accounting for less than 5% of the observed changes.

**Figure 4:**
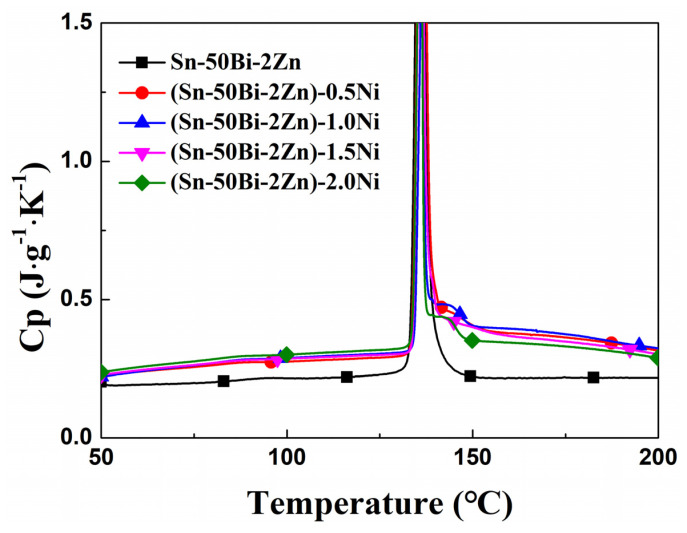
Comparison of the specific heat of alloy samples.

The variation curve of the specific heat capacity of the alloy is presented in Figure 4, while Table 4 displays the average specific heat capacity across different physical states. The arithmetic mean of the three measurements is taken as the approximate true value of the specific heat capacity test result. The specific heat capacity of the (Sn–50Bi–2Zn)–xNi alloy in its solid state exhibits an increase with the augmentation of nano Ni particle content, reaching a maximum value of 0.27 J·g^−1^·K^−1^. This rise can be attributed to the high specific heat of the Ni particles themselves. As the temperature rises, the specific heat of the matrix alloy in the liquid state remains relatively stable. However, in samples with the inclusion of nano Ni particles, the specific heat of the liquid alloy experiences a significant increase, with a maximum value of 0.38 J·g^−1^·K^−1^, indicating a favorable bonding between the second-phase nano Ni particles and the liquid alloy. Nevertheless, this phenomenon follows a trend of initially increasing and subsequently decreasing, potentially due to improved dispersibility at lower nano Ni particle contents. As the content increases, the nano nickel particles aggregate, leading to a reduction in the micro interface of the alloy matrix and a decrease in the impact effect. This conclusion aligns with the findings obtained from the microstructure analysis.

**Figure 5:**
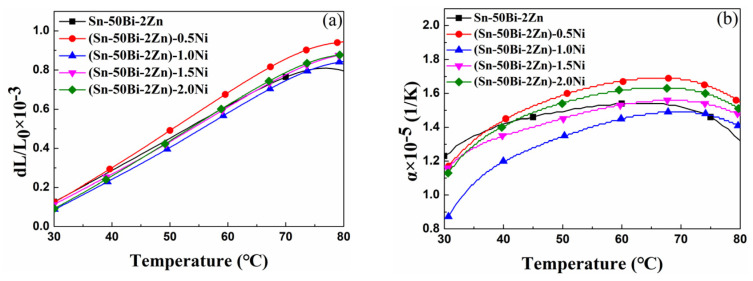
Experimental results of thermal expansion of alloy samples: (**a**) relative elongation, (**b**) linear thermal expansion coefficient.

The thermal expansion curve of the (Sn–50Bi–2Zn)–xNi alloy is presented in Figure 5. The results depicted in Figure 5a demonstrate a linear variation in the elongation of the alloy, with values of 0.618 × 10^−3^, 0.859 × 10^−3^, 0.746 × 10^−3^, 0.777 × 10^−3^, and 0.788 × 10^−3^, respectively. The inclusion of nano-sized Ni particles enhances the elongation properties of the alloy. Conversely, Figure 5b illustrates that the linear expansion coefficient of the alloy gradually increases with rising temperatures until reaching a plateau, with values of 14.3, 16.7, 14.5, 15.3, and 16.2 × 10^−6^/°C. The addition of nano-sized Ni particles has minimal impact on the thermal expansion coefficient of the base alloy. Since the disparity between the thermal expansion coefficient of Sn–50Bi–2Zn and the linear expansion coefficient of the Ni element is marginal, and the Ni content only ranges from 0.5% to 2.0%, the thermal expansion coefficient remains relatively stable.

**Figure 6:**
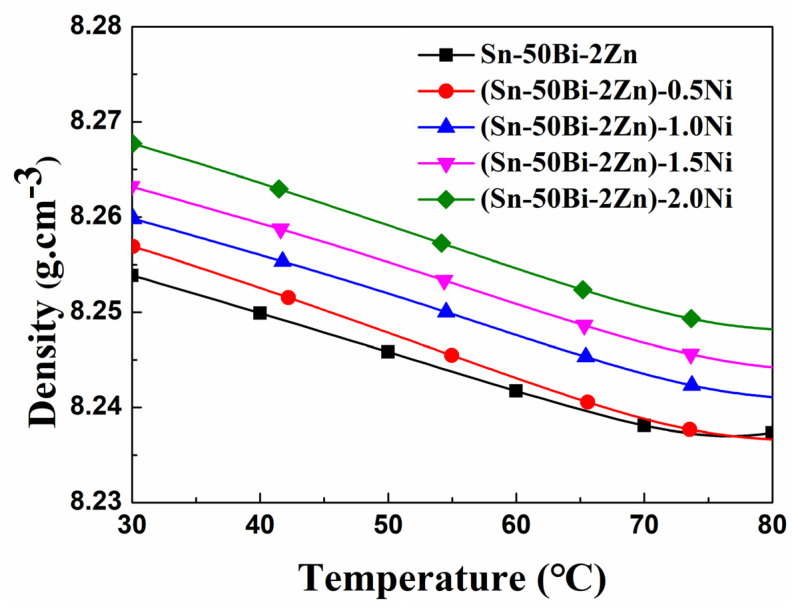
Density-temperature curve.

The densities of (Sn–50Bi–2Zn)–xNi alloys at room temperature were found to be 8.253, 8.256, 8.259, 8.263, and 8.268 g/cm^3^, respectively. The data are the average of 10 measurements. These results indicate that the addition of a small number of nano nickel particles does not significantly enhance the density of the alloy. Figure 6 illustrates the density curve obtained using Formula (1), showing that the densities of all alloys decrease as the temperature increases. This phenomenon can be attributed to a slight expansion of the alloys’ volume with increasing temperature, while the mass remains constant, resulting in a minor reduction in the alloys’ density.

The thermal diffusivities of (Sn–50Bi–2Zn)–xNi alloys were determined using the laser flash method and are presented in Table 5. The data in the table demonstrate that the inclusion of nano Ni particles leads to a reduction in the thermal diffusivity of the alloy. Notably, (Sn–50Bi–2Zn)–1.0Ni exhibits the lowest thermal diffusivity of 14.61 mm^2^/s. This decrease can be attributed to the formation of additional micro-interfaces within the alloy matrix by the nanoparticles. During the heat transfer process, both free electrons and phonons interact with metal atoms and encounter scattering at interfaces and various defects, thereby generating thermal resistance. Consequently, the thermal diffusivity of the alloy matrix decreases.

**Figure 7:**
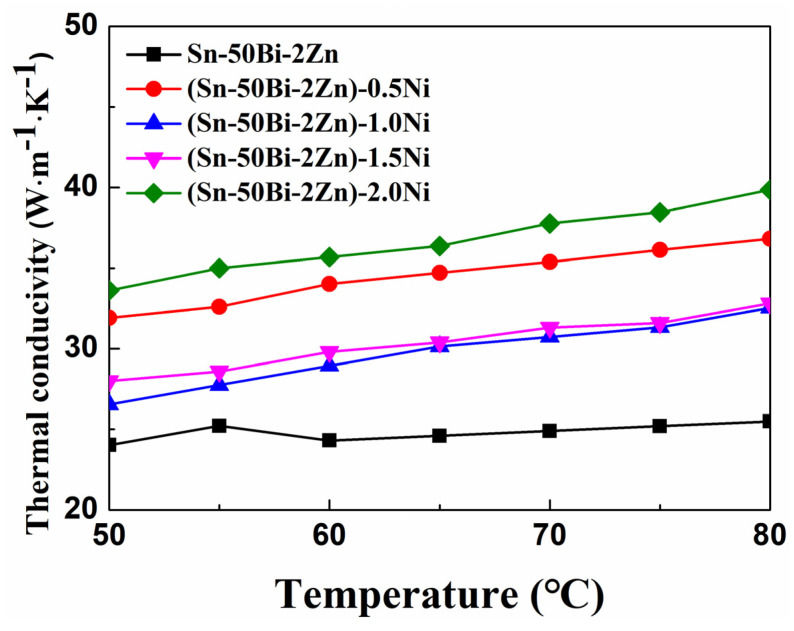
Temperature dependence of the thermal conductivity.

Figure 7 shows the thermal conductivity of the (Sn–50Bi–2Zn)–xNi alloy in the solid state, as calculated using Formula (2). The thermal conductivity of (Sn–50Bi–2Zn)–2.0Ni is higher than that of other alloys. The addition of nickel nanoparticles as the second phase particles, due to the collision and scattering effects, leads to the reduction in thermal diffusivity, which affects the heat transfer efficiency of the alloy, but with the increase in the specific heat capacity of the alloy, this effect is gradually offset. The results of the thermal conductivity of the alloy in the solid state show that the addition of nanoparticles can increase the specific heat capacity of the base alloy, but it is difficult to detect the thermal diffusivity of the alloy in the liquid state, so it is impossible to accurately obtain the thermal conductivity data of the liquid alloy.

Currently, extensive research has been conducted within the academic community to elucidate the mechanism through which nanoparticles enhance specific heat [25,26,27,28,29,30]. This enhancement may arise from the specific heat effect inherent to the nanoparticles themselves, the formation of an adsorption layer between the nanoparticles and the materials, or the presence of interfacial thermal resistance between the two materials, thereby increasing the specific heat of the matrix material. In this study, we propose that the enhancement of thermal conductivity in the solid state may be due to the modification of eutectic phase morphology by nanoparticles and the induction of ultrafine grains in the composite alloy [31], as shown in Figure 2a,e,i, which changes the contribution ratio of electron and phonon heat transfer in the alloy. The enhancement of the specific heat of composite alloys in the liquid state may be due to the formation of an ordered state between the second-phase solid nanoparticles and the liquid molten metal. More direct microthermal analysis requires improved observation methods, including 3ω-methods to decouple thermal conductivity, heat capacity, and thermal diffusivity, and to conduct more refined theoretical modeling. Nevertheless, this new discovery is of great significance for the development and application of nanoparticle-enhanced heat transfer and storage alloys with excellent strength and high thermal conductivity.

## 4. Conclusions

This study found that adding nano Ni particles can improve the thermal properties of the matrix alloy. When the content of Ni particles exceeds 1.0 wt.%, the dispersibility deteriorates. On the other hand, nanoparticles can alter the morphology of eutectic phases and induce ultrafine grains in composite alloys. In addition, it significantly increased the specific heat capacity of the alloy in the liquid state, which may be due to the formation of an ordered state between the second-phase solid nanoparticles and the liquid molten metal. With the increase in temperature, the destruction of these ordered states requires additional heat, which leads to the increase in specific heat capacity. More direct microthermal analysis requires breakthroughs in experimental methods and instruments, as well as more refined micro theoretical modeling.

## Figures and Tables

**Table 1:** Composition ratio of the alloy samples.

Samples	Designed Compounds	XRF Results (wt.%)			
		Sn	Bi	Zn	Ni
A1	Sn–50Bi–2Zn	48.29	49.57	2.14	0
A2	(Sn–50Bi–2Zn)–0.5Ni	47.86	49.71	1.91	0.52
A3	(Sn–50Bi–2Zn)–1.0Ni	47.65	49.52	1.87	0.97
A4	(Sn–50Bi–2Zn)–1.5Ni	47.63	48.99	1.86	1.52
A5	(Sn–50Bi–2Zn)–2.0Ni	47.77	48.42	1.83	1.98

**Table 2:** The atomic percentage composition of the marked points in the graph.

Elemental Composition	A	B	C	D	E	F	G
Sn	0	93.24	20.20	59.52	22.84	16.88	48.61
Bi	100	4.86	16.35	0	67.62	12.27	14.35
Zn	0	1.90	63.45	40.48	0	50.82	5.83
Ni	0	0	0	0	9.54	22.16	31.21
Phase composition	Bi-rich	Sn-rich	Zn-rich	Sn + Zn	Bi + Ni	NiZn	Sn + Ni

**Table 3:** The DSC data of alloys.

Samples	Melting Temperature/(°C)			Melting Enthalpy/(J·g^−1^)
	Onset	Peak	End	ΔH_m_
A1	135.49	138.15	139.47	47.62
A2	134.48	136.44	138.26	47.07
A3	134.02	135.79	136.94	46.45
A4	134.04	135.66	137.51	45.24
A5	133.91	135.73	136.65	48.14

**Table 4:** Specific heat of alloys at different temperatures.

Samples	The Average Specific Heat Capacity (J·g^−1^·K^−1^)	
	Solid State	Liquid State
A1	0.21	0.22
A2	0.25	0.36
A3	0.25	0.38
A4	0.26	0.35
A5	0.27	0.33

**Table 5:** Heat diffusion coefficient of the alloys.

Samples	Thermal Diffusivity (mm^2^/s)
A1	18.19
A2	17.18
A3	14.61
A4	14.73
A5	16.94

## Data Availability

Data is contained within the article.
